# Adherence to facemask use in public places during the autumn–winter 2020 COVID-19 lockdown in Greece: observational data

**DOI:** 10.1186/s12991-022-00386-2

**Published:** 2022-03-09

**Authors:** Konstantinos N. Fountoulakis, Joao Breda, Marianna P. Arletou, Anastasios I. Charalampakis, Maria G. Karypidou, Konstantina S. Kotorli, Christina G. Koutsoudi, Eleftheria S. Ladia, Calypso A. Mitkani, Vasiliki N. Mpouri, Anastasia C. Samara, Aikaterini S. Stravoravdi, Ioannis G. Tsiamis, Aphrodite Tzortzi, Maria A. Vamvaka, Charikleia N. Zacharopoulou, Panagiotis E. Prezerakos, Sotirios A. Koupidis, Nikolaos K. Fountoulakis, Eva Maria Tsapakis, Anastasia Konsta, Pavlos N. Theodorakis

**Affiliations:** 1 Department of Psychiatry, School of Medicine, Faculty of Health Sciences, Aristotle University of Thessaloniki, 6, Odysseos str, 55535 Thessaloniki, Greece; 2 WHO Athens Quality of Care Office, WHO Regional Office for Europe, Athens, Greece; 3 School of Medicine, Faculty of Health Sciences, Clinical Mental Health, Aristotle University of Thessaloniki, Thessaloniki, Greece; 4 Department of Nursing, Laboratory of Integrated Health Care, University of Peloponnese, Tripoli, Greece; 5 Occupational & Environmental Health Sector, Public Health Policy Department, School of Public Health, University of West Attica, Athens, Greece; 6 Faculty of Medicine, Medical University of Sofia, Sofia, Bulgaria; 7 Agios Charalampos Mental Health Clinic, Heraklion, Crete Greece; 8 Health Policy, WHO Regional Office for Europe, Athens, Greece

**Keywords:** Facemasks, COVID-19, Health behavior

## Abstract

**Background:**

Wearing facemasks is of proven efficacy as a public health protective measure against COVID-19. Currently there are no observational data concerning the wearing of facemasks and the adherence to guidelines concerning their handling.

**Methods:**

Registration of the way passers-by were wearing facemasks at 26 different locations of five major cities in Greece. The results were correlated with the rate of COVID-19 deaths in the region.

**Results:**

In total, 119,433 passers-by were registered, 57,043 females (47.8%) and 62,390 males (52.2%). From the total sample, 81.1% were wearing the mask properly, 10.8% had their nose out, 6.2% were wearing it under the jaw, and 1.9% had no mask at all . There was a significant difference between males and females concerning any use of mask. Inappropriate use of was correlated with COVID-19 death rate in the studied region.

**Conclusion:**

Our findings suggest that under conditions of mandatory wearing and in central locations of major cities, during walking, proper use of masks is suboptimal, but still contributes with some protection. Fear and risk perception seem to be strong factors contributing to adherence to proper mask wearing.

## Background

The first to use a face mask for sanitary reasons with a modern theory in mind were Johann Mikulicz, head of the surgery department of the University of Breslau (now Wroclaw, Poland), and Paul Berger in Paris in 1897 [1]. Today it is known that when a contagious person coughs or sneezes, droplets containing infectious particles could be released [2, 3]. The importance of suppressing this potential source of pathogens was shown during the Manchurian plague of 1910–11, the influenza pandemic of 1918–19, and more recent 2003 and 2009 influenza pandemics [1].

Airborne transmission is probably the dominant way of COVID-19 transmission [4–9]; hence, wearing face masks is among the most efficacious, cheapest, and easiest measures to control it [10, 11]. Other measures include ventilation, air cleaning devices, and similar [12, 13]. Eventually, masks were recommended by government authorities and their usefulness was supported by scientific data [14–37], but unfortunately early in the course of the pandemic conflicting opinions and recommendations were made. This caused much confusion [38], and probably led to lower adherence with mask wearing or improper use of them. This in turn could have led to an at least partially cancel of their protective effect [39–41].

Many governments have implemented mandatory facemasks even in public open spaces. However, the efficacy of such measure depends on the material the mask is built upon as well as on the way masks are used. Also, as most public health sanitary measures, the protective effect of mask wearing tends to be more collective than personal; it demands that the overwhelming majority of the population will adopt the measure and will adhere to the rules and guidelines.

At the time of the study, there seems to be much uncertainty as well as a discrepancy. The uncertainty concerned the degree of adoption of the measure by the population. There are some poll data, but they are based on self-report online questionnaires and reports are anecdotal. Until now, there are no observational data concerning facemasks wearing of and the adherence to guidelines concerning its handling. Also, there are no data on the quality of facemasks and the materials they are made of.

The extent to which public health measures and appropriate health-related behaviors are adopted by the general public is a matter of great importance and should be the focus of mental health professionals. This kind of behaviors seem to stem from psychological characteristics and also follow the rules of mass psychology. In the future, managing of problematic attitudes and behaviors could be a prime target when facing a pandemic or other kinds of crises and mass destruction, and therefore, the studying these phenomena during the current pandemic is essential.

This study aimed at collecting observational data about the ways of wearing facemask in public, by observing and counting passers-by walking on the street under real-life situations. The outcome should be considered as reflecting the adherence to protective measures in general. The observational conditions chosen are considered to reflect the condition under which adherence is the maximum (walking in public, not socializing, mandatory use, monitoring by the police).

## Methods

The aim of this study was to register the rates of passers-by wearing facemasks, their sex, and age group as well as the way they were wearing the mask, during a period of obligatory mask wearing in open spaces.

This study was an initiative by the School of Medicine under the auspice of the Rector of the Aristotle University of Thessaloniki in collaboration with the Panhellenic Medical Association. It took place between 18 November and 13 December 2020 and was approved by the Ethics committee, School of Medicine, Faculty of Health Sciences, Aristotle University of Thessaloniki Greece.

**Fig. 1:**
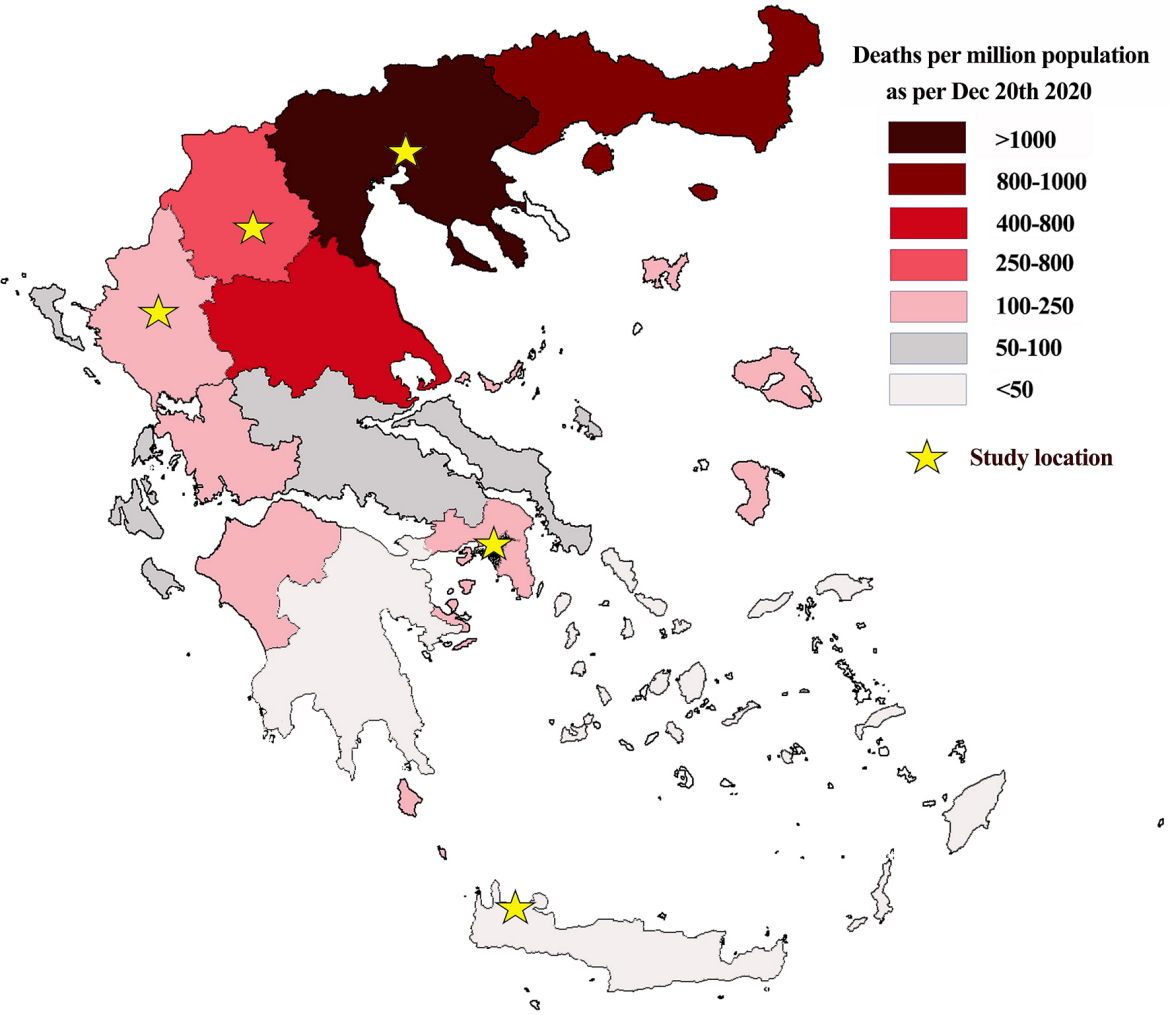
Map of Greece with the five centers and COVID-19 mortality rates per administrative region

Data collection was performed by fourteen researchers at 26 different locations of five big cities in Greece, both in the morning and in the afternoon for periods of three hours each time. The five cities were in areas of the country with different standardized COVID-19 mortality rates (Fig. 1).

The registration included gender and estimated age group. Field workers registered the way the person was using the mask as follows: (a) correctly, (b) nose out, (c) under the jaw, and (d) not at all. Age was classified into five age groups: 15–17 years, 18–30, 31–45, 46–60, and > 60. Age was registered according to the estimation by the rater; no contact with passers-by was initiated.

Only passers-by were registered; hence, there was no information collected for people standing, visiting any kind of shop, discussing with others, socializing etc., in order to have a homogenous result. The reason why the person was not wearing his/her mask properly (e.g., drinking or smoking while walking) was not taken into account. There were no questions concerning the material of the mask or the details of its use (cleaning, touching, etc.).

In cases passers-by were too many to register, the rater was instructed to register as many as possible in a consecutive but not selective sequence, and to record the instance. No such an instance was reported.

The composition of the study sample was compared with population data from the Greek Statistics Authority (ELSTAT) from www.statistics.gr, and expected numbers were calculated.

Official data on COVID-19 death rates until December 20, 2021, published by the Ministry of Health (Fig. 1) were used for the five locations.

The statistical analysis included Chi-square for the testing of frequencies and Spearman correlation coefficients were used to test for the relationship between mask wearing and COVID-19 deaths in the region at the time of data collection.

## Results

In total, 119,433 passers-by were registered, 57,043 females (47.76%) and 62,390 males (52.24%).

From the total sample, 96,818 (81.06%) were wearing the mask properly, 12,906 (10.81%) had their nose out, 7451 (6.24%) were wearing it under the jaw, and 2258 (1.89%) had no mask at all. In detail the results are shown in Table 1.

**Table 1:** Facemasks wearing by sex and age group and COVID-19 death rates in the five regional districts of the country

	N	Nose out	Jaw	Not at all	Total	%	Nose out	Jaw	Not at all	Jaw + not at all	Regional COVID-19 death rate	
	Correctly					Correctly					Males	Females
Males	48,228	7630	5003	1529	62,390	77.30	12.23	8.02	2.45	10.47		
< 18	1943	553	328	233	3057	63.56	18.09	10.73	7.62	18.35		
18–30	11,062	1877	1360	310	14,609	75.72	12.85	9.31	2.12	11.43		
30–45	11,885	1775	1302	475	15,437	76.99	11.50	8.43	3.08	11.51		
45–60	13,127	2037	1239	362	16,765	78.30	12.15	7.39	2.16	9.55		
> 60	10,211	1388	774	149	12,522	81.54	11.08	6.18	1.19	7.37		
Females	48,590	5276	2448	729	57,043	85.18	9.25	4.29	1.28	5.57		
< 18	2013	425	235	86	2759	72.96	15.40	8.52	3.12	11.63		
18–30	13,659	1626	807	167	16,259	84.01	10.00	4.96	1.03	5.99		
30–45	14,561	1570	777	271	17,179	84.76	9.14	4.52	1.58	6.10		
45–60	12,230	1160	486	155	14,031	87.16	8.27	3.46	1.10	4.57		
> 60	6127	495	143	50	6815	89.90	7.26	2.10	0.73	2.83		
Athens	19,322	1985	1565	873	23,745	81.37	8.36	6.59	3.68	10.27	272.53	156.35
Thessaloniki	57,168	7399	4494	1165	70,226	81.41	10.54	6.40	1.66	8.06	1330.13	840.71
Ptolemaida	8721	1416	633	122	10,892	80.07	13.00	5.81	1.12	6.93	500.78	472.13
Ioannina	9460	1193	646	91	11,390	83.06	10.47	5.67	0.80	6.47	205.10	122.75
Chania	2147	913	113	7	3180	67.52	28.71	3.55	0.22	3.77	51.84	19.08

From all the age groups, only females aged over 45 years old, manifested correct use of mask above 85%.

Chi-square test revealed a significant difference between males and females concerning any use of mask (chi-square = 1353.606, df = 3, *p* < 0.0001), and this was true in all sub-groups: correct vs not-correct use of the mask (Chi-square = 1205.583, df = 1, *p* < 0.0001), correct or nose out vs jaw or not at all (Chi-square = 958.039, df = 1, *p* < 0.0001), and any use vs not at all (Chi-square = 220.942, df = 1, *p* < 0.0001).

There was also a difference among the age groups both for males (Chi-square = 796.015, df = 16, *p* < 0.0001) and for females (Chi-square = 2439.912, df = 16, *p* < 0.0001).

Both for gender and for age groups, the differences were significant for all paired comparisons.

The comparison of the study population with the general population of the country revealed an excess of males and young females in our sample (Table 2).

**Table 2:** Comparison of the study sample gender and age distribution with the general population

	General population	%	Observed in study sample		Expected	Difference % from expected
	N		N	%	N	
Males	4,459,864	48.06	62,390	52.24	57,397.54	8.70
15–17	273,727	2.95	3057	2.56	3522.811	– 13.22
18–30	599,106	6.46	14,609	12.23	7710.372	89.47
31–45	1,191,268	12.84	15,437	12.93	15,331.38	0.69
46–60	1,087,118	11.71	16,765	14.04	13,990.99	19.83
> 60	1,308,645	14.10	12,522	10.48	16,842	– 25.65
Females	4,820,236	51.94	57,043	47.76	62,035.46	– 8.05
15–17	263,919	2.84	2759	2.31	3396.584	– 18.77
18–30	587,150	6.33	16,259	13.61	7556.501	115.17
31–45	1,194,031	12.87	17,179	14.38	15,366.94	11.79
46–60	1,177,657	12.69	14,031	11.75	15,156.21	– 7.42
> 60	1,597,479	17.21	6815	5.71	20,559.23	– 66.85

Spearman correlation coefficients between COVID-19 death rates per region and rates of mask wearing (data shown in Table 1) were below 0.3 for all correlations except from those concerning ‘mask under the jaw’ and ‘not at all,’ for both male and female death rates, which were above 0.7. No correlation was significant eventually due to the small size of the dataset; therefore, only the analysis of numerical values is possible.

## Discussion

During 2020, almost 95% of the world population was living in countries where the use of facemasks in public places was mandatory at some time and for prolonged periods of time, in order to contain the COVID-19 pandemic. There are specialized medical facemasks and cloth facemasks with the latter being washable and reusable and aiming to be used by the general population [42, 43].

Although, eventually, masks were recommended by government authorities and their usefulness was supported by scientific data [14–37], early in the course of the pandemic conflicting opinions and recommendations were made, leading to long-lasting confusion [38]. Improper wearing or mishandling of masks could at least partially cancel their protective effect [39–41]. The extent of this problem in the real world is unknown; however, models of the protective effect under different conditions have been proposed [40, 41]. Until now there was no report on the actual adoption of mask wearing especially in European countries, which is a determining factor for the real-world success of such a measure of public health. To our knowledge, the only study concerning the efficacy of facemasks in open spaces was underpowered and its methodology conceived masks as if they are an independent measure of self-protection [44], which is one of the most faulty ways of studying their usefulness. An additional problem was that around the world, the overall quality of these masks is relatively unknown [45].

To our knowledge, the current is one of the first studies in the literature to register the real-world use of facemasks under mandatory conditions. Unfortunately, our findings do not agree with the positive speculation for an excellent adoption of facemasks by the public in front of a deadly pandemic [46]. They suggest that in the middle of the second wave of the pandemic, under conditions of mandatory wearing, and in central places of major cities, during walking, proper use of masks is on average around 80% and only in females aged over 45 the percentage climbs above 85%. It is reasonable to assume that in almost all other conditions the adherence would be significantly lower. We should add the unknown specifications of mask fabric and the unknown adherence to guidelines on how to handle the mask. These findings, together with their most probable interpretation, suggest that the measure of mask wearing may be working but at a suboptimal level, and in combination with a reasonable lockdown they probably lead to a 50% reduction in death rates [40, 41], but it proved to be insufficient to prevent the third wave of the pandemic which eventually hit the country.

The second finding of this study was that percentage of people not wearing masks correlates with both male and female COVID-19 death rates in the region. This implies that the determining factor for adherence behavior is fear and risk perception rather than information, or at least it constitutes a very strong element [47–50].

The third important finding was related with sex and age composition of the study sample in comparison to the general population. While the composition of circulating people under normal conditions is unknown, the results of the current study suggest that middle aged and older females (all aged > 45) have probably reduced their time outside home. In males this was also evident maybe only in those above 60 years of age. It is to be noted, however, that the age was roughly estimated, so there is some kind of uncertainty concerning this variable and this should be considered as a limitation of the current study.

Taking all these together, the conclusion should be that the use of mask by the public is suboptimal but still probably partially efficacious. This should be considered together with the finding that cases and deaths were steadily going downwards even after more than a month after the end of the data collection of the current study.

Mask wearing is culturally strange in western societies in contrast to east Asian ones who consider this kind of behavior as social and collective responsibility [51]. Still, the pandemic has changed radically western views but the extent of this is largely unknown and of unknown influence on the end-efficacy [52]. There are reports suggesting better adherence to facemask wearing with increasing age [53] and with higher rates in females (54). Both these are confirmed by our findings, which additionally suggest the relationship with age is linear. The gender difference is impressive; no male age group has higher rate from any female group except from the very young aged < 18 (Table 1).

Apart from sociocultural elements, psychological characteristics are important in the adoption of health-related behaviors, and in this specific situation probably the cognitive style is of high importance. Emotions probably play a significant role and anger together with high level of anxiety was prominent in the general population during the period of the gathering of the data for the current study.

## Conclusions

The results of the current study

- suggest that mandatory mask wearing produced suboptimal results, but still probably exerted a significant effect on COVID-19 death rate;
- provide a possible explanation why almost all countries around the world were eventually obliged to utilize some form of lockdown; and
- suggest that difference in adherence between genders is at least one of many contributing factors in the observed difference between males and females concerning morbidity and mortality from COVID-19.

## Data Availability

The data are available upon request from the first author.
